# Congenital limb deficiency in Japan: a cross-sectional nationwide survey on its epidemiology

**DOI:** 10.1186/s12891-018-2195-3

**Published:** 2018-07-27

**Authors:** Hiroshi Mano, Sayaka Fujiwara, Kazuyuki Takamura, Hiroshi Kitoh, Shinichiro Takayama, Tsutomu Ogata, Shuji Hashimoto, Nobuhiko Haga

**Affiliations:** 1 Department of Rehabilitation Medicine, The University of Tokyo Hospital, Tokyo, Japan; 2 Department of Rehabilitation Medicine, Graduate School of Medicine, The University of Tokyo, Tokyo, Japan; 3 Department of Orthopaedic Surgery, Fukuoka Children’s Hospital, Fukuoka, Japan; 4 Department of Orthopedic Surgery, Nagoya University Graduate School of Medicine, Nagoya, Japan; 5 Department of Orthopaedic Surgery, National Center for Child Health and Development, Tokyo, Japan; 6 Department of Pediatrics, Hamamatsu University School of Medicine, Hamamatsu, Japan; 7 Department of Hygiene, Fujita Health University School of Medicine, Toyoake, Japan

**Keywords:** Congenital limb deficiency, Epidemiology, Cross-sectional study, Nationwide survey, Birth prevalence

## Abstract

**Background:**

Congenital limb deficiency is a rare and intractable disease, which impairs both function and appearance of the limbs. To establish adequate medical care, it is necessary to reveal the actual conditions and problems associated with this disease. However, there have been no extensive epidemiological surveys in Japan addressing this disease. This is the first nationwide epidemiological survey of congenital limb deficiency in this country.

**Methods:**

With the cooperation of epidemiology experts, we performed a two-stage nationwide survey to estimate the number of patients with congenital limb deficiency and reveal basic patient features. We targeted orthopaedic surgery, paediatric, and plastic surgery departments. Hospitals were categorized according to the institution type and the number of hospital beds; hospitals were randomly selected from these categories. We selected 2283 departments from a total 7825 departments throughout Japan. In this study, we defined congenital limb deficiency as partial or total absence of the limbs, proximal to the proximal interphalangeal joint of the fingers/lesser toes or interphalangeal joint of the thumb/great toe. We distributed the first survey querying the number of initial patient visits from January 2014 to December 2015. Targets of the second survey were departments that reported one or more initial patient visits in the first survey.

**Results:**

In the first survey, 1767 departments responded (response rate: 77.4%). Among them, 161 departments reported one or more initial patient visits. We conducted the second survey among these 161 departments, of which 96 departments responded (response rate: 59.6%). The estimated number of initial visits by patients with congenital limb deficiency was 417 (95% confidence interval: 339–495) per year in 2014 and 2015. The estimated prevalence of congenital limb deficiency in Japan was 4.15 (95% confidence interval: 3.37–4.93) per 10,000 live births. The sex ratio was 1.40. Upper limbs were more affected than lower limbs.

**Conclusions:**

We revealed the estimated number of initial patient visits per year and birth prevalence of congenital limb deficiency in Japan. Our results will contribute to establishing the disease concept and grades of severity of congenital limb deficiency.

**Electronic supplementary material:**

The online version of this article (10.1186/s12891-018-2195-3) contains supplementary material, which is available to authorized users.

## Background

In developed countries, paediatric limb deficiencies are primarily congenital, and result from various causes and phenotypes. Causal factors include environmental insults, such as owing to chemicals and pharmaceuticals, and chromosomal or genetic defects. Thalidomide, warfarin, valproic acid, and phenytoin are well known as causal drugs of limb deficiencies [1]. Some genetic factors associated with hypoplastic limb defects and split hand/split foot malformation have been identified [2]. The prevalence of congenital limb deficiencies is reportedly 4.91 per 10,000 live births in South America (1967–1992) [3], 5.5 per 10,000 total births in Alberta, Canada (1980–2012) [4], and 6.9 per 10,000 total births in Northern Netherlands (1991–2010) [5]. According to the International Clearinghouse for Birth Defects Monitoring Systems (ICBDMS), the prevalence of congenital limb deficiency in Japan is reportedly 3.81 per 10,000 total births (2007–2011) [6]. Although some breakdown has been reported (transverse: 0.29; pre axial: 0.90; post axial: 0.25; intercalary: 1.04; mixed: 0.90; unspecified: 0.43 per 10,000 total births), distinctions between upper or lower limbs, laterality, and other details (e.g., level of transverse deficiency) have not been demonstrated. In addition, it seems difficult to make precise diagnoses at birth in some patients. Regarding anomalies of the upper limbs in the Japanese population, Ogino et al. reported incidences and details such as affected side, associated anomalies, and family history [7]; however, these anomalies consisted mostly of deformities without limb deficiency or reduction such as trigger finger, polydactyly, camptodactyly and clasped thumb, and the situation for deficiency only was uncertain.

Congenital limb deficiency is a rare and intractable disease, and these patients require consecutive care from birth, through the stages of growth, and up to adulthood. However, in Japan, medical care and education systems have been inadequate for affected children. In considering therapeutic approaches, it is necessary to include both the function and appearance of the defect. Treatment approaches vary according to the type of deficiency. For example, children with congenital unilateral transverse upper limb deficiencies are often prescribed prostheses [8]. For radial deficiency, surgeries such as wrist centralization on the ulna and reconstruction of the thumb are the treatment choices [9]. In proximal focal femoral deficiency, surgeries including amputation or rotationplasty may be considered to optimise prosthetic fitting and function [10]. There is debate regarding which surgical approach to choose in certain situations. For example, for Jones type 1a tibial deficiency, one surgical approach is tibial centralization surgery (Brown’s procedure), to reconstruct the function of the knee-joint [11]. Another procedure is knee disarticulation to fit a prosthesis [12]. Regarding fibular dysplasia, one approach is foot reconstruction followed by limb-lengthening surgery, to preserve the affected limb; another is Syme’s amputation procedure, followed by a prosthetic fitting [13, 14].

In Japan, nationwide epidemiological surveys have been performed in patients with various intractable diseases [15] such as familial Mediterranean fever [16], Moyamoya disease [17, 18], Churg–Strauss syndrome [19], adult Still’s syndrome [20], Sjögren’s syndrome [21], paediatric acquired demyelinating syndrome [22], and pancreatitis [23, 24]; however, congenital limb deficiency has not been studied in detail. To provide better evidence-based medical care, it is necessary to establish the concept of congenital limb deficiency and its grades of severity. In this study, we aimed to clarify the actual condition of congenital limb deficiency in Japan.

## Methods

With the cooperation of the Research Committee on the Epidemiology of Intractable Diseases of Japan, we conducted a two-stage postal survey according to the *Nationwide Epidemiological Survey Manual of Patients with Intractable Diseases* (2nd edition 2006, Ministry of Health, Labour and Welfare of Japan).

This nationwide survey consisted of a first survey, in which the number of patients was estimated, and a second survey, in which basic clinical features were investigated. To achieve a high response rate, it was important to use a simple questionnaire. We confined the first survey to only a query of the number of patients who completed an initial visit. In the second survey, we requested the ages, symptoms, diagnostic details, associated anomalies, family histories of patients, and the previous hospitals and referral to other hospitals as the minimum of demographic and clinical information.

A list of all hospitals in Japan was obtained from the Research Committee on the Epidemiology of Intractable Diseases of Japan. The departments of orthopaedic surgery, paediatrics, and plastic surgery were extracted and subjected to stratified random sampling. Hospitals were categorized according to the institution type and number of hospital beds. Departments were randomly selected from these hospital categories; sampling rates were determined as approximately 5, 10, 20, 40, 80, and 100% for the stratum of general hospitals with 20–99 beds, 100–199 beds, 200–299 beds, 300–399 beds, 400–499 beds, and 500 or more beds, respectively. As the departments that most commonly treat patients with congenital limb deficiency, departments belonging to “the Japanese Association of Children’s Hospitals and Related Institutions” and “Rehabilitation Centers for Children with Physical Disabilities” were classified as “special stratum”, and all of these were selected for the survey. Other departments that could not be classified under any of the three departments above (i.e., hospitals in which medical care was provided by a specially associated group of multi-departments) were classified as “other special stratum,” and were selected for the survey.

From this selection, 2283 departments (995 orthopaedic surgery departments, 812 paediatric departments, 472 plastic surgery departments), and four special departments were selected from a total of 7825 departments throughout Japan. In May 2016, we distributed the first survey querying the number of initial patient visits from January 2014 to December 2015. In this study, we defined congenital limb deficiency as partial or total absence of the limbs, proximal to the proximal interphalangeal joint of the fingers/lesser toes or interphalangeal joint of the thumb/great toe. We focused on deficiencies that cause relatively severe impairment and disability, for which treatment approaches have been discussed; therefore, we excluded deficits of the middle and distal phalanges. Polydactyly, syndactyly, and malformation or shortening of the limbs without deficit were also excluded from this study.

The targets of the second survey were departments that reported one or more initial patient visits in the first survey. In September 2016, we distributed the second survey querying patient details. In the second survey, patients who were ineligible, those who were not eligible because of their diagnosis or the time of their initial visit, and duplicate patients who were reported by two or more departments, were determined. Patients were identified as duplicated if their information (age, sex, diagnosis, and referral) was consistent among two or more departments. For duplicate patients, we included the data of their latest initial visit at the most recent, to obtain a more precise diagnosis and more detailed information. If a patient had visited more than one hospital during a short time, it is reasonable to think that the first institution saw the necessity for a more precise diagnosis or specialized medical treatment, such as a surgical procedure or prosthesis, and consulted a specialized department and that the most recent institution provided them with medical care. We identified the department for the date of the most recent initial visit as the actual institution that provided medical treatment at that time.

### Statistics

The method used to estimate the number of patients is as follows [25, 26]:

Let *n* be the number of all departments, and *n*_*i*_ be the number of departments with a number of targeted patients *i* (*i* = 0, 1, …). Let *N* be the number of responding departments and *N*_*i*_ be the number of responding departments with a number of patients *i* (*i* = 0, 1, …). *N* and *N*_*i*_ were obtained from the survey, and {*N*_*i*_} followed a multi-hypergeometric distribution under the assumption of random response and under the condition that N is fixed.

Let *a* be the total number of patients; note that *a* = ∑*i*∗*n*_*i*_. The estimate of *a* was calculated as.

$$ a\hat{\mkern6mu} =\sum i\ast {N}_i\ast n/N. $$

The approximate 95% confidence intervals for *a* were calculated as.

$$ \left(a\hat{\mkern6mu} -1.96\ s,a\hat{\mkern6mu} +\kern0.37em 1.96\ s\right), $$

where *s* is an estimate of standard error of *a^* and is given to be.

$$ s=\sqrt{\frac{\sum {i}^2\ast \frac{N_i}{N}-{\left(\sum i\ast \frac{N_i}{N}\right)}^2}{n-1}\kern0.28em \ast \kern0.28em {n}^3\kern0.28em \left(\frac{1}{N}-\frac{1}{n}\right)\kern0.28em }. $$

The number of patients in some strata was estimated using the above method. Let k be the number of strata, *a*_*1*_*^*, *a*_*2*_*^*, …, *a*_*k*_*^* be the estimated numbers of patients in some strata, and *s*_*1*_, *s*_*2*_, *…, s*_*k*_ be their standard errors. The approximate confidence intervals for the sum of the number of patients in the strata were calculated as.

$$ \left(a.\hat{\mkern6mu} -1.96\ s.,a.\hat{\mkern6mu} +\kern0.37em 1.96\ s.\right), $$

where *a*. = *a*_*1*_ ^  + *a*_*2*_ ^  + … + *a*_*k*_^ and $$ s=\sqrt{{s_1}^2+{s_2}^2+\dots +{s_k}^2} $$.

Using the results of the first survey, the number of patients and 95% confidence intervals were estimated using the above procedure. Additionally, we corrected these using the ineligibility rate and duplication rate from the second survey. To obtain the final estimates and confidence intervals, we multiplied their values in the first survey by “1 − ineligibility rate” and “1 − duplication rate”, respectively.

Statistical analysis was performed using JMP® Pro 13.0.0 (SAS Institute Japan) and *p* < 0.05 was considered significant.

## Results

### Estimation of the number of patients

Table 1 summarizes the results of the first survey and shows the corrected number of patients with the results of the second survey. Of 2283 departments selected from the 7825 departments in Japan, 1767 responded to the first questionnaire concerning the number of patients with initial visits who had congenital limb deficiency: the response rate for the first survey was 77.4%. Details of the number of all, surveyed, and responding departments are shown in Additional file 1. Among them, 161 departments reported one or more patients with an initial visit. We then conducted the second survey among these 161 departments. Ninety-six departments responded to the second questionnaire concerning demographic and clinical information; the response rate for the second survey was 59.6%. Of these 96 departments, whereas a total number of 534 patients had an initial visit in the first survey, 491 patients with initial visits were recorded in the second survey. This difference of 43 patients was considered to represent ineligible patients and those excluded by the target departments. Some departments reported the reasons for exclusion as follows: intermixing of patients whose initial visit did not fall within the target period or did not fit the diagnosis (e.g., patients with acquired limb defects) in the first survey. Of the 491 patients, we excluded 38 ineligible patients and 42 duplicated patients; finally, 411 patients were eligible for inclusion in the analysis of demographic and clinical information. Of the 38 ineligible patients, 27 did not fall within the target period and 11 did not fit the diagnosis (4 patients had deficits of only the middle and/or distal phalanx, 4 had brachydactyly without deficits, 2 had constriction band without deficits, and 1 patient had polydactyly). The ineligibility rate was 15.2% and the duplication rate was 9.3%.

**Table 1:** Number of total, surveyed, and responded departments, and estimated number of patients

Strata	Total Number of Departments	Number of Surveyed Departments	Sampling rate (%)	Number of Responded Departments	Response Rate (%)	Estimated Number of Patients	95% Confidence Interval		
First Survey									
Orthopedic surgery									
University hospital	133	133	100.0	120	90.2	104	87	–	121
General hospital									
≧500 beds	201	201	100.0	155	77.1	25	16	–	33
400–499 beds	213	170	79.8	135	79.4	0	0	–	0
300–399 beds	343	137	39.9	104	75.9	7	0	–	14
200–299 beds	391	79	20.2	59	74.7	0	0	–	0
100–199 beds	1006	100	9.9	75	75.0	40	0	–	96
≦99 beds	1950	96	4.9	64	66.7	0	0	–	0
Special stratum	79	79	100.0	65	82.3	290	230	–	351
Subtotal	4316	995	23.1	777	78.1	466	381	–	551
Pediatrics									
University hospital	125	125	100.0	106	84.8	24	18	–	29
General hospital									
≧500 beds	189	189	100.0	152	80.4	25	18	–	31
400–499 beds	193	154	79.8	128	83.1	8	3	–	12
300–399 beds	302	121	40.1	93	76.9	97	0	–	230
200–299 beds	297	60	20.2	51	85.0	12	0	–	26
100–199 beds	486	48	9.9	33	68.8	0	0	–	0
≦99 beds	786	39	5.0	29	74.4	0	0	–	0
Special stratum	76	76	100.0	50	65.8	15	6	–	25
Subtotal	2454	812	33.1	642	79.1	180	47	–	314
Plastic surgery									
University hospital	89	89	100.0	68	76.4	47	35	–	60
General hospital									
≧500 beds	157	157	100.0	107	68.2	129	6	–	252
400–499 beds	125	100	80.0	75	75.0	30	10	–	50
300–399 beds	127	52	40.9	38	73.1	7	0	–	14
200–299 beds	126	25	19.8	20	80.0	0	0	–	0
100–199 beds	186	19	10.2	13	68.4	0	0	–	0
≦99 beds	222	11	5.0	8	72.7	0	0	–	0
Special stratum	19	19	100.0	15	78.9	46	28	–	63
Subtotal	1051	472	44.9	344	72.9	259	132	–	385
Other special stratum	4	4	100.0	4	100.0	179	179	–	179
Total	7825	2283	29.2	1767	77.4	1084	881	–	1287
Total (per year)						542	441	–	643
Second Survey									
Total (per year) ^a^						417	339	–	495
Total (per 10,000 live births) ^b^						4.15	3.37	–	4.93

^a^The estimated number of patients and 95% confidence interval was corrected with ineligible rate and duplication rate

^b^We estimated with the assumption that the number of live birth was 1.005million

We estimated that the number of initial visits of patients with congenital limb deficiency between 2014 and 2015 was 417 per year, (95% confidence interval 339–495). Considering that at the initial visit, most patients were aged 0 years, and that the number of live births in Japan was 1.004 million in 2014 and 1.006 in 2015, we estimated the prevalence of congenital limb deficiency with the assumption that the number of live births was 1.005 million (average of 2014 and 2015). The estimated prevalence was 4.15 (95% confidence interval 3.37–4.93) per 10,000 live births.

### Sex ratio

Among 411 patients reported in the second survey, there were 240 males and 171 females; the sex ratio was 1.40. Assuming an equal sex ratio (50% male, 50% female), a chi-square test yielded χ^2^ = 11.58, and *p* = 0.0007. This result suggests that males have a significantly higher prevalence of congenital limb deficiency than females.

### Age

Table 2 summarizes the age of patients at their initial visit. Congenital limb deficiencies develop during the foetal stage, or before birth. The age reflects the patients’ access to medical care. It is necessary to consider that in cases of duplication, we included data for the date of the most recent initial visit to a department. A total 276 (67.2%) patients visited the hospital at age 0 years. The number of patients with initial visits decreased with advancing age.

**Table 2:** Patient age at the initial visit

Age	Number of Patients	%
0	276	67.2
1	39	9.5
2	22	5.4
3	18	4.4
4	9	2.2
5	10	2.4
6	6	1.5
7	5	1.2
8	4	1.0
9	3	0.7
10	2	0.5
≥11	12	2.9
Unknown	5	1.2
Total	411	100

### Affected limbs

Among 411 patients reported in the second survey, 275 (66.9%) had one affected limb, 83 (20.2%) had two, 23 (5.6%) had three, and 30 (7.3%) had four affected limbs. There were 275 patients (66.9%) with only the upper limbs affected, 75 (18.2%) with only lower limbs affected, and 61 (14.8%) with both upper and lower limbs affected.

The 411 patients had a total of 630 affected limbs among them. Four limbs had different classifications of deficiency (a total of 634 limb deficiencies) and all four affected limbs had coexisting proximal femoral focal deficiency and fibular dysplasia. Affected limbs were as follows: 218 (34.6%) were right upper limbs, 224 (35.6%) were left upper limbs, 105 (16.7%) were right lower limbs, and 83 (13.2%) were left lower limbs.

### Classification of limb deficiency

Table 3 summarizes the classification of limb deficiency. For upper limbs deficiencies, transverse deficiency was the most prevalent (45.5%), followed by longitudinal deficiency(31.2%). For lower limbs deficiency, longitudinal deficiency was the most prevalent (37.0%), followed by transverse deficiency(31.8%). For longitudinal deficiency, whereas there was not much difference between tibial deficiency (33 limbs) and fibular deficiency (37 limbs) in the lower limbs, radial deficiency (110 limbs) was more prevalent than ulnar deficiency (27 limbs) in the upper limbs. For transverse deficiency, both upper limbs and lower limbs had similar tendencies in that distal deficiency comprised a large proportion and proximal deficiency was less frequent. The prevalence rates of intercalary, central, and other finger column deficiency were similar for both upper and lower limbs.

**Table 3:** Classification of congenital limb deficiency

	Upper limbs			Lower Limbs			Total	
		Number of deficiencies	%		Number of deficiencies	%	Number of deficiencies	%
Longitudinal	Radial deficiency	110	24.9	Tibial deficiency	33	17.2		
	Ulnar deficiency	27	6.1	Fibular deficiency	37	19.3		
	Not classifiable	1	0.2	Not classifiable	1	0.5		
	Subtotal	138	31.2	Subtotal	71	37.0	209	33.0
Transverse	Shoulder, Upper arm, Elbow	16	3.6	Hip, Thigh, Knee	2	1.0		
	Forearm, Wrist	41	9.3	Lower leg, Ankle	4	2.1		
	Hand	141	31.9	Foot	54	28.1		
	Unknown	3	0.7	Unknown	1	0.5		
	Subtotal	201	45.5	Subtotal	61	31.8	262	41.3
Intercalary	Phocomelia	5	1.1	Proximal femoral focal deficiency	7	3.6		
	Subtotal	5	1.1	Subtotal	7	3.6	12	1.9
Central	Split hand	75	17.0	Split foot	32	16.7		
	Subtotal	75	17.0	Subtotal	32	16.7	107	16.9
Other finger column deficiency		23	5.2		21	10.9	44	6.9
Total		442	100		192	100	634	100

### Associated anomalies

Coexisting disorders with relatively high prevalence (over 1%), were cardiac anomaly, vertebral anomaly, renal anomaly, anal atresia, hypospadias, cleft lip/palate and developmental disorders (autism spectrum syndrome or attention deficit hyperactivity disorder) (10.2, 3.6, 2.4, 2.4, 2.2, 1.2, and 1.0% respectively). Eleven patients had chromosomal abnormality: 3 had 17p-related abnormalities (duplication, microduplication and deletion), 4 had trisomy 21, 1 had trisomy 18, 1 had 48 XXXX, and 2 patients unspecified chromosomal abnormalities. Seventeen patients were diagnosed with or suspected of having the following specific syndromes, associations or sequences: 5 patients had VATER association, 4 had Poland syndrome (1 patient concomitantly had Moebius syndrome), 2 had Cornelia de Lange syndrome, and 1 each, had Apert syndrome, CHARGE syndrome, FATCO syndrome, KOSAKI syndrome, Leri-Weill syndrome, Nager syndrome, Silver-Russell syndrome, facioauriculovertebral sequence, and autosomal dominant epidermolysis bullosa dystrophica (we excluded amniotic bands syndrome). Approximately 66.4% of patients had no coexisting disorders.

### Family history

Seventeen (4.1%) patients had a family history of congenital limb deficiency, and 165 (40.1%) had no such history. The family histories of 229 (55.7%) patients were unknown. The extent of family history was left to the discretion of each department.

Table 4 shows the details of 17 patients with a positive family history of congenital limb deficiency. The breakdown of these 17 patients was as follows: 9 with split hand deformity; 3 with ulnar deficiency; 2 each with upper limb transverse deficiency, split foot, and tibial deficiency; and 1 each with radial deficiency, lower limb transverse deficiency, and other finger column deficiency. Four patients had multiple types of deficiencies: 1 each with split hand and foot, split hand and tibial deficiency, ulnar deficiency and split foot, and ulnar deficiency and tibial deficiency. Most columns in the questionnaire requiring information about the limb deficiency of family members were left blank; therefore, we could not identify whether the type of deficiencies were the same for these patients.

**Table 4:** Details of patients with a positive family history of congenital limb deficiency

Child	Deficiency of the limb	Associated anomaly	Relation of the relative	Limb anomaly of the relative
1	split hand of right upper limb	none	sibling	(not reported)
2	split hand of right upper limb	none	sibling	(not reported)
3	split hand of right upper limb	none	sibling	(not reported)
4	split hand of right upper limb	none	sibling	(not reported)
5	split hand of both upper limbs and split foot of both lower limbs	none	sibling	(not reported)
6	radial deficiency of left upper limb	Fanconi’s anemia	sibling	(not reported)
7	ulnar deficiency of right upper limb	none	sibling	(not reported)
8	transverse deficiency at the level of hand of right upper limb	none	sibling	(not reported)
9	split hand of right upper limb	none	mother	anomalies of radius and ulna
10	split hand of right upper limb	none	father	split hand
11	split hand of both upper limbs	none	father	split hand
12	split hand of left upper limb and tibial deficiency of both lower limbs	none	granduncle	split hand
13	split foot of right lower limb	none	someone other than sibling	(not reported)
14	ulnar deficiency of both upper limbs and split foot of both lower limbs	Leri-Weill syndrome	mother	Madelung deformity, and Leri-Weill syndrome
15	ulnar deficiency of both upper limbs and tibial deficiency of both lower limbs	none	mother and grandmother	upper limb anomaly
16	transverse deficiency at the level of hand of right upper limb	none	relative on the mother’s side	toe defect
17	transverse deficiency at the level of foot of both lower limbs	none	father and grandfather on the father’s side	anomaly of fifth finger

## Discussion

This is the first study providing the detailed epidemiology of congenital limb deficiency in Japan. We achieved a higher response rate (77.4%) for the first survey than the 34–74% of similar studies conducted in Japan [16–24]. This may reflect an interest in limb deficiency by orthopaedic surgeons, paediatricians, and plastic surgeons. The estimated prevalence of congenital limb deficiency in Japan of the present study was similar to that of previous studies in other countries [3–5].

Whereas the duplication rate in this study seemed reasonable, the ineligibility rate seemed somewhat high. Similar surveys have reported low duplication rates, and the ineligibility rates varied by disease (2.3–13.2%) [17, 18, 20, 21]. One possible reason for the high ineligibility rate in this study was the inconsistent responses by some departments regarding the number of patients who had completed an initial visit and the total number of patients. In similar surveys in Japan, the number of patients and the number of initial patient visits, or only the number of patients were usually requested. In this study, to achieve a high response rate, we only queried the number of initial patient visits, which might have confused respondents.

The prevalence value was also similar to that reported in Japan by ICBDMS [6]; however the classification of congenital limb deficiency was different, especially for intercalary deficiency. In this study, intercalary deficiency included phocomelia and proximal femoral focal deficiency. In the ICBDMS report, a definition of “intercalary” was not available and the “limb reduction defects” did not include “central reduction.” These differences in definitions may have influenced the results of our survey. Our results were more similar to those of a study in the Northern Netherlands that reported fewer number of intercalary reduction defects than transverse, longitudinal, and central reduction defects [5].

The sex ratio was 1.40 in this study, which is similar to a study in Alberta, Canada, including 434 male and 344 female patients [4].

For most patients, the initial visit to the most recent institution was made at age 0 years, and the number of initial patient visits decreased with advancing age. Most congenital limb deficiencies are noted at birth, even if they are not diagnosed in detail; however, therapeutic interventions are rarely required in the neonatal period. No radical surgeries or therapies are normally carried out. It is recommended that the first fitting of prostheses commence at about age 6–9 months in children with limb deficiencies [27]; however, prosthetic therapy often begins later in Japan. In particular, prostheses for children with congenital upper limb deficiencies have not been sufficiently prescribed [28]. In our study, we assumed that there were patients who were untreated or were only followed-up without therapy and that some of them visited a specialized hospital for surgeries or prosthetic therapy or sought a second opinion during childhood or later in life.

Upper limbs were more affected than lower limbs in the present study. Previous studies showed the same tendency [4, 5]. Commonly associated anomalies in our survey were cardiac anomaly, vertebral anomaly, renal anomaly, anal atresia, hypospadias, and cleft lip/palate. The study in the Northern Netherlands also indicated cardiovascular anomalies and urinary tract anomalies as anomalies that are frequently present with reduction defects [5]. The specific syndromes, associations, or sequences diagnosed or suspected in more than one patient were already known to be associated with congenital limb deficiency. Considering the data thus far, we feel certain about the validity of the present results.

Seventeen (4.1%) patients had a family history of congenital limb deficiency; however, the family history of 229 (55.7%) patients was unknown. Possible reasons for the substantial number of patients with unknown family histories include the following:

(1) The respondent may have completed the survey in detail, but the family history was truly unknown.

(2) Although respondents reported that there was no family history, the extent was insufficient (e.g., only parents were known to have no limb deficiency, but this information for grandparents was unknown), and the respondent thought it was inadequate to consider as not-existence.

(3) The respondents were too busy to complete the survey in detail.

If patients with “unknown” family histories were excluded, the prevalence of patients with a family history of congenital limb deficiency was 9.3%. It seems reasonable that the prevalence was estimated at 4–10% in this study. Most patients with a family history were those with split hand (a portion of these patients were associated with split foot or tibial deficiency). We speculate that split hand might have a stronger relationship with genetic factors than other limb deficiencies, such as transverse deficiency, of which constriction band is one cause. This is consistent with a study that identified the genetic factor associated with split hand/foot malformation and Gollop-Wolfgang complex (association with split hand and tibial deficiency) among Japanese patients [29]; this is also in keeping with the fact that most split hand/foot malformation, including Gollop-Wolfgang complex, present an autosomal dominant pattern of inheritance [2]. Another speculation is that patients with split hand procreate more frequently than those with other limb deficiencies; further study of this relationship is warranted.

The present survey was cross-sectional and involved analyses of demographics, and clinical information such as prevalence and classification. To promote the development of therapeutic approaches and social support of patients with congenital limb deficiency, further surveys based on the results of this study are required. A longitudinal survey is expected to reveal the actual conditions and challenges throughout life among these patients, such as education, employment, marriage, and reproduction.

### Limitations

Although the response rate for the first survey was high (77.4%), the ineligibility rate (15.2%) in the second survey seemed somewhat high. The main reason for exclusion was that the time of the initial visit did not correspond with the target period. In addition, there were some patients who did not fit the appropriate definition of congenital limb deficiency used in this study (defined as partial or total absence of the limbs proximal to the proximal interphalangeal joint of fingers/lesser toes or interphalangeal joint of the thumb/great toe). The disease concept of congenital limb deficiency has not been established in Japan. The estimates number in the present study may have been overestimated or underestimated, or may have changed in accordance with the definition used for this disease.

## Conclusions

We estimated the prevalence of congenital limb deficiency in Japan to be 4.15 (95% confidence interval 3.37–4.93) per 10,000 live births. Upper limbs were more affected than lower limbs. For upper limbs, transverse deficiency was the most prevalent, followed by longitudinal deficiency. For lower limbs, longitudinal deficiency was the most prevalent, followed by transverse deficiency. We also revealed information of the sex ratio, age at initial visit, associated anomalies, and family history. Although we believe that our results will contribute to establishing the disease concept and grades of severity of this disease, further surveys, especially longitudinal ones, will be needed to promote the development of therapeutic approaches and social support for patients with congenital limb deficiency.

## Additional file

Additional file 1: Numbers of Total, Surveyed, and Responded Departments, and Breakdown of Responded Departments by Numbers of Reported Patients. This is the raw data used in the first survey of this study. (XLSX 12 kb)
